# Albendazole and ivermectin for the control of soil-transmitted helminths in an area with high prevalence of *Strongyloides stercoralis* and hookworm in northwestern Argentina: A community-based pragmatic study

**DOI:** 10.1371/journal.pntd.0006003

**Published:** 2017-10-09

**Authors:** Adriana Echazú, Marisa Juarez, Paola A. Vargas, Silvana P. Cajal, Ruben O. Cimino, Viviana Heredia, Silvia Caropresi, Gladys Paredes, Luis M. Arias, Marcelo Abril, Silvia Gold, Patrick Lammie, Alejandro J. Krolewiecki

**Affiliations:** 1 Instituto de Investigaciones en Enfermedades Tropicales, Universidad Nacional de Salta-Sede Regional Orán, San Ramón de la Nueva Orán, Salta, Argentina; 2 Consejo Nacional de Investigaciones Científicas y Técnicas (CONICET), Buenos Aires, Argentina; 3 Facultad de Ciencias Naturales, Cátedra de Química Biológica, Universidad Nacional de Salta, Salta, Argentina; 4 Gerencia Sanitaria, Hospital Juan Domingo Perón, Tartagal, Salta, Argentina; 5 Secretaria de Nutrición y Alimentación Saludable, Ministerio de Salud Pública de la Provincia de Salta, Salta, Argentina; 6 Departamento de Programas y Proyectos, Fundación Mundo Sano, Buenos Aires, Argentina; 7 Division of Parasitic Diseases and Malaria, Centers for Disease Control and Prevention, Atlanta, Georgia, United States of America; University of Queensland, AUSTRALIA

## Abstract

**Background:**

Recommendations for soil-transmitted helminth (STH) control give a key role to deworming of school and pre-school age children with albendazole or mebendazole; which might be insufficient to achieve adequate control, particularly against *Strongyloides stercoralis*. The impact of preventive chemotherapy (PC) against STH morbidity is still incompletely understood. The aim of this study was to assess the effectiveness of a community-based program with albendazole and ivermectin in a high transmission setting for *S*. *stercoralis* and hookworm.

**Methodology:**

Community-based pragmatic trial conducted in Tartagal, Argentina; from 2012 to 2015. Six communities (5070 people) were enrolled for community-based PC with albendazole and ivermectin. Two communities (2721 people) were re-treated for second and third rounds. STH prevalence, anemia and malnutrition were explored through consecutive surveys. Anthropometric assessment of children, stool analysis, complete blood count and NIE-ELISA serology for *S*. *stercoralis* were performed.

**Principal findings:**

STH infection was associated with anemia and stunting in the baseline survey that included all communities and showed a STH prevalence of 47.6% (almost exclusively hookworm and *S*. *stercoralis*). Among communities with multiple interventions, STH prevalence decreased from 62% to 23% (p<0.001) after the first PC; anemia also diminished from 52% to 12% (p<0.001). After two interventions *S*. *stercoralis* seroprevalence declined, from 51% to 14% (p<0.001) and stunting prevalence decreased, from 19% to 12% (p = 0.009).

**Conclusions:**

Hookworm’ infections are associated with anemia in the general population and nutritional impairment in children. *S*. *stercoralis* is also associated with anemia. Community-based deworming with albendazole and ivermectin is effective for the reduction of STH prevalence and morbidity in communities with high prevalence of hookworm and *S*. *stercoralis*.

## Author summary

Soil-transmitted helminth (STH) infections are a relevant public health problem in resource restricted settings due to their potential to perpetuate poverty, since chronic infections are associated with learning and grow impairment in children and reduced productivity in adults. The current strategy for STH control gives a key role to preventive chemotherapy of risk groups (preschool and school age children and women of childbearing age) with anthelmintic drugs. The drugs recommended for regular deworming are albendazole or mebendazole. This strategy does not target *Strongyloides stercoralis*, an STH resistant to the recommended drugs in single doses. The efficacy of ivermectin, alone or in combination, for the treatment of *Strongyloides stercoralis* infection has been reported in controlled trials. We conducted a pragmatic study aiming to assess the effectiveness of community based preventive chemotherapy with albendazole plus ivermectin for the control of STH prevalence and morbidity, in endemic communities of Northwestern Argentina. We found high baseline prevalence of hookworm and *Strongyloides stercoralis* and significant nutritional and hematological morbidity associated with these infections. After three rounds of preventive chemotherapy with albendazole and ivermectin we observed a significant decline in the prevalence of infection and in the prevalence and severity of morbidity.

## Introduction

Soil-transmitted helminth (STH) infections are the most prevalent Neglected Tropical Diseases (NTD) worldwide affecting over 2 billion people. Four nematode species (*Ascaris lumbricoides*, *Trichuris trichiura*, *Necator americanus* and *Ancylostoma duodenale*) are the most common STH infections of humans [1,2]. Due to their common biological characteristics and risk factors, geographic overlap and anthelmintic treatment of choice, the recommendations from the World Health Organization (WHO) for STH control target the four species together [3]. *Strongyloides stercoralis* is an STH with similar distribution but with some distinctive characteristics regarding its diagnosis and therapy that have prevented its inclusion in the current guidelines for STH control; however, it could be targeted with updated comprehensive control strategies [4,5].

STH morbidity is of public health concern because of the population affected, the high prevalence in low income countries and the long lasting consequences of the infection, all contribute to the economic impact of the disease and perpetuation of poverty [6]. Chronic STH infection has been associated with cognitive impairment in school age children (SAC) and negative impact on motor and language development of preschool age children (PSAC) [7–9]. Negative effects of STH infection on nutritional status of children have also been described such as stunting, reduced weight gain and specific micronutrient deficiencies (i.e. iron and vitamin A) [10]. A major consequence of STH infection is iron-deficiency anemia, of significant relevance in infants and pregnancy outcomes. STH infections and other tropical infectious diseases, such as schistosomiasis and malaria, are implicated in the etiology of iron-deficiency anemia in lesser-developed countries [11,12]. Among them, hookworm infection has been strongly associated with the development of iron-deficiency anemia, due to chronic intestinal blood loss [13].

Comprehensive STH control strategies include health education, improvements in water, sanitation and hygiene, and Preventive chemotherapy (PC) through mass drug administration (MDA) of albendazole or mebendazole [14,15]. Implementation of PC for school age children (SAC), the group targeted by the current recommendations, has shown to be effective in reducing worm burden and STH prevalence as much as in improving nutritional status and anemia [16,17]. However, critical groups of the population like PSAC and women of reproductive age are not directly reached by this approach and would benefit if another strategy for deworming such as community-based PC were applied [9,18]. The use of a drug active against *S*. *stercoralis* (ivermectin) along with albendazole or mebendazole would enhance the PC effectiveness in the control of *S*. *stercoralis* and *T*. *trichiura* and would carry additional benefits such as the reduction in the prevalence of scabies and impetigo [5,19–21].

Argentina has a heterogeneous prevalence of STH infection, with areas of high prevalence in the north. Previous studies in Salta province, in the Northwest, showed a cumulative prevalence of STH near 50%, with preponderance of *S*. *stercoralis* (20–48%) and hookworm (20–45%) [22,23]. Anemia is a public health problem that affects 18% of the Argentinian population, particularly in the northwest, where 38% of the PSAC and 19% of women between 10 and 49 years old are anemic [24,25].

The aim of the present study was to assess the effectiveness of a community based PC program against STH, using a combination of albendazole and ivermectin. For that purpose, we compared the results of fecal, blood and anthropometric surveys carried out before each PC intervention through a longitudinal community based operational research. The effectiveness of the intervention was monitored through the evaluation of STH cumulative prevalence of the participant communities and variation in morbidity indicators such as anemia and nutritional status of children. The study was part of a larger project with the objective to incorporate a program of PC for STH control into the regular activities of the public primary health care system in high prevalence regions of Salta province, Argentina.

## Methods

### Study design

Community-based pragmatic non-randomized trial conducted in Tartagal, Salta province, Argentina between August 2012 and May 2015.

### Ethics statement

All participants selected for surveillance provided written informed consent prior to the study and parents/guardians provided informed consent on behalf of minor participants. The research protocol and the informed consent forms were approved by the Bioethics Committee of the Colegio de Médicos de la Provincia de Salta and by the Bioethics Committee of the Faculty of Health Sciences at the Universidad Nacional de Salta (FWA registered committee). The anthelmintic drugs were used according to currently approved and recommended regimens.

### Study population

All members of six communities from Tartagal were invited to participate in the study. A community is defined as the group of people with a common ethnic origin that lives in neighboring households and shares a unique community leader; generally including a group of around 100 households. Four of the communities enrolled in the study were peri-urban: Lapacho Alto, Kilometro 6, Las Moras and Lapacho I; and two communities were urban: Pablo Secretario and Tapiete, These communities are served by the Provincial primary health care system; therefore, trained public health personnel (“sanitary agents”) visited each household every three months. The communities were selected by the local public health authorities based on the sanitary risk indicators collected in the census the year before the study started. Communities with report of malnutrition in PSAC, child or maternal mortality of preventable causes or known STH prevalence above 50% were enrolled. All these communities also share similar water and sanitation conditions [23] and are homogeneous in their economic status, most of the families have a low monthly income that comes from informal and transitory jobs of a single breadwinner and from small economic benefits from the government. A sample was recruited for stool and blood surveillance, using stratified random selection with community as the stratification factor and household as the unit for choice. Inhabitants of the selected households, of any age and gender, were asked to provide a stool and blood samples prior to deworming. Since this is a population-based trial, the subjects recruited for surveillance were not necessarily the same individuals at baseline and follow-up. Nevertheless, the random selection strategy and sample size of the survey group were similar throughout the study. The number of stool samples collected within the survey group was 397 at baseline; 130 at first follow up and 181 at second follow up. Blood samples were 409 at baseline; 156 at first follow up and 165 at second follow up.

### Intervention

All the participants were offered anthelmintic therapy, independent of their age (age ranging from 1 to 91 years old) or recruitment to the survey group. Two anthelmintic drugs in single doses were used simultaneously, albendazole 400mg tablets, dosed at 1 tablet (half the dose in children 12 to 24 months of age) and ivermectin 6mg tablets dosed at 200 micrograms/kg; produced by GlaxoSmithKline and ELEA Argentina respectively. The drugs were administered according to the following inclusion and exclusion criteria. Inclusion criteria: 1) Permanent resident of the enrolled community; 2) Willing to take anthelminthic drugs. Exclusion criteria: 1) Age < 12 months (albendazole); 2) Body weight < 15 kg (ivermectin); 3) Confirmed or suspected pregnancy, contraindication for albendazole in the first trimester and for ivermectin in any trimester; 4) Women breast-feeding new born babies, contraindication for ivermectin in the first week of puerperium; 5) Rejection to take anthelminthic drugs; 6) Known allergy to albendazole or ivermectin; 7) Taking an alternative anthelminthic therapy at the moment of the intervention.

PC was carried out by health personnel and members of the research team who distributed the drugs through intensive deworming campaigns in the communities (house by house) and local schools. Each campaign lasted 4 days per community and was carried out sequentially, due to operative limitations. During the deworming campaigns, all the participants who met all the inclusion and none of the exclusion criteria received the anthelmintic therapy. Passive pharmaco-vigilance activities were carried at the local tertiary hospital (Hospital Juan D. Peron) and the respective sanitary posts of ambulatory and emergency services were aware of the interventions. The first intervention took place in August 2012 and the last one in May 2015; during that period, some communities were treated once and others received three rounds of PC with an interval of 9 to 16 months between rounds. Stool and blood surveillance were performed at baseline and follow-up (before each round of PC). The results of the interventions were entered in the data base for each participant as: treated (if the subject was administered one or two drugs); excluded (if both drugs were contraindicated); absent (if the subject was not found at home during the deworming round or the household could not be found); death; migration (if the subject moved from the community before the intervention) and rejection (if the subject did not want to take the anthelminthic drugs when offered). Coverage was calculated through the formula: n people treated/n people eligible for treatment and eligibility was calculated as: total population–(excluded + death + migrants).

### Measurements

The following data were gathered for the study: a) baseline socio-demographic information; b) baseline and follow-up anthropometric data; c) baseline and follow-up stool results; d) baseline and follow-up hematologic results, and e) baseline and follow-up serologic results for *S*. *stercoralis*. The methods used for data collection are detailed below:

a) Socio-demographic assessment: public health census forms include data about each household of the community, collected through direct observation by the sanitary agent, along with individual demographic information for each inhabitant of the household. Demographic information was entered in the study database using the sanitation and drinking-water ladders designed by WHO/UNICEF for sanitary census [26].

b) Nutritional survey: during the deworming visits, members of the research team registered weight and height of children from one to fifteen years-old. Weight was measured using a standard electronic scale in kilograms and grams. Height was measured with a steel tape while the child was standing near to a wall and registered in centimeters. In order to make the results comparable, population based sampling was used; the number of baseline height and weight observations was above the minimum sample of 400 suggested by WHO for nutritional surveys. Results were compared to the NCHS/WHO reference population and the expected ranges of standard deviations of the anthropometric indicators were considered to control the accuracy of the measurements. Anthropometric data was analyzed through WHO Anthro and WHO Anthro Plus softwares (Department of Nutrition, WHO) to calculate relevant Z-scores. Weight, height, Weight-for-Age z-score (WAZ), Height-for-Age z-score (HAZ) and Weight-for-Height z-score (WHZ) of each participant child at baseline (before the first PC round) and follow-up (before second and third PC rounds) were registered. Children were classified as stunting (HAZ <-2 SD from the international reference median value); underweight (WAZ <-2 SD from the international reference median value) or wasting (WHZ <-2 SD from the international reference median value) according to the WHO recommendations on z-scores interpretation [27].

c) Parasitological surveillance: a single fresh stool sample without preservatives was collected from each participant of the survey group. Sterile stool containers and instructions were distributed house by house, collected the following morning and analyzed within 24 hours of collection in a reference laboratory. Five parasitological techniques were used: sedimentation/concentration; agar plate culture; Harada-Mori filter-paper culture; Baermann concentration of charcoal-cultured fresh stool, and McMaster egg counting method as described elsewhere (28,29). If the sample volume was insufficient to perform all methods, concentration technique was prioritized due to its overall higher sensitivity in preliminary studies [28]. A high level of certainty in the distinction between hookworm and *S*. *stercoralis* larvae in the culture techniques was assured by the different incubation time of the methods (24 hours for Baermann and 7 days for Harada-Mori), adapted to each specie life cycle, and by the long experience of the technicians who observed and supervised the microscopic exams. The findings of the different methods were grouped and entered in the database as positive if at least one method was positive or negative if all the methods were negative, for each STH species. McMaster´s results were recorded as egg per gram (EPG). The stool survey was performed at baseline and before each PC campaign.

d) Hematological surveillance: participants of survey group had 5 mL blood drawn through venipuncture. A complete blood count was performed using a SYSMEX automated hematology analyzer KX 21N. The results of Hemoglobin value (Hgb), white blood cell count (WBC) and eosinophil relative count were registered. Subjects were classified as anemic or not anemic using the Hgb thresholds to define anemia according to sex and age set by WHO/UNICEF [12]. Absolute eosinophil count was calculated and eosinophilia was defined as absolute eosinophil values >500 cells/mm^3^ [29].

e) Blood samples were centrifuged and an aliquot of serum was preserved frozen at –20°C and analyzed with the in-house enzyme-linked immunosorbent assay (NIE-ELISA) method for the diagnosis of *S*. *stercoralis*. NIE-ELISA detects IgG antibodies against recombinant NIE antigen of *S*. *stercoralis* L3 larvae, as has been described previously [30,31]. Patient’s sera were tested in duplicate and compared to a standard positive IgG curve run on each plate. The averages of duplicate results were calculated and corrected for background reactivity (no serum added).

### Outcomes

The primary outcome was STH prevalence. The cumulative and species specific STH prevalence before and after deworming was compared to monitor the effectiveness of community-based PC. Secondary outcomes were indicators of morbidity potentially due to STH infection such as anemia, eosinophilia and nutritional impairment.

The study was analyzed in two phases: i) baseline assessment that included a description of the study population targeted with PC and an exploration of the survey group at the individual level, searching for associations of STH infection with nutritional and hematological findings; and ii) longitudinal assessment to evaluate the impact of PC on population-related parameters in the communities where repeated PC was carried out.

### Statistical analysis

Sample size was estimated considering a predicted prevalence of 50%, a confidence level of 95%, and a design effect of 2. Sample size calculation was based on observing a specific reduction of 10% or more in STH prevalence; the estimated sample size was n = 190.

Continuous quantitative measures evaluated at different time points were described using proportions with 95% confidence intervals (95% CI); means with standard deviations (SD) and medians with interquartile ranges (IQR). Comparisons between infected and uninfected people at baseline and between pre and post intervention parameters were carried out using T test and Mann-Withney U test. Significant associations between STH infection and morbidity indicators were explored through stratified bivariate analysis and, afterwards, adjusted through multivariate logistic regression models; statistical significance was assessed by Chi-square test with 95% significance. Correlation between continuous quantitative measures was explored through linear regression tests of Pearson´s or Spearman´s (according to the underlying distribution).

All data was entered in Microsoft Access 11.5 (Microsoft, Redmond, WA) with an Epi Info 3.5.4 (CDC, Atlanta, GA) view. Duplicate data entry was performed by trained collaborators. The analysis was performed with EPIDAT 3.1 (PAHO, Washington, DC) and R 3.1.1 (The R Foundation for Statistical Computing, GNU General Public License).

## Results

**Fig 1:**
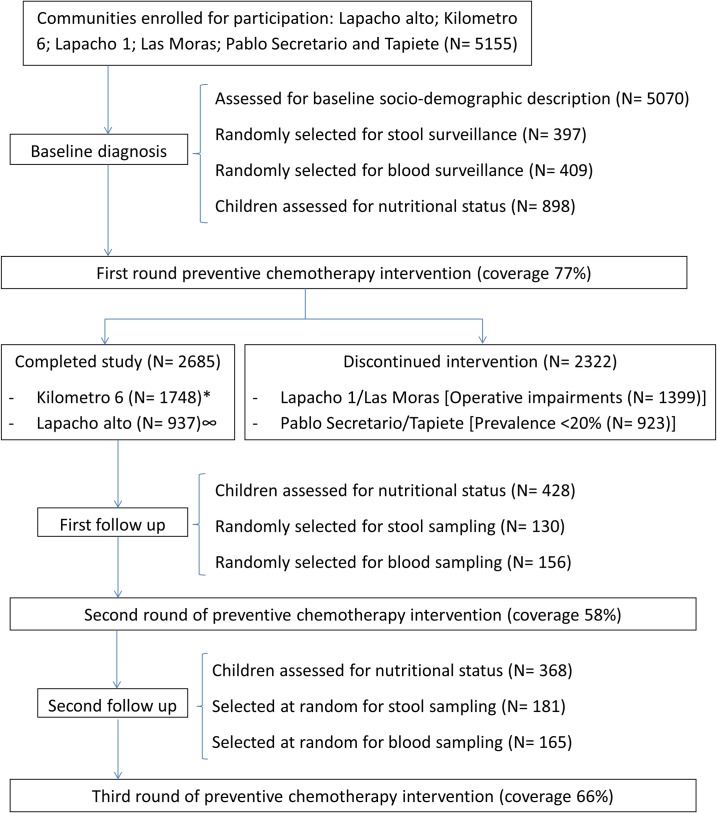
Flow diagram of the participants and size of surveillance samples enrolled along the study. *In Lapacho Alto community 9 months went by between the first and the second intervention and 14 months between second and third intervention. ∞In Kilometro 6 the period between first and second intervention was 9 months and between second and third intervention was 20 months.

A total of 5070 inhabitants of six communities were enrolled in the study, 400 of them were recruited in the survey group at baseline. Two communities: Kilometro 6 and Lapacho Alto received three deworming interventions, therefore a baseline and two follow up surveillances were carried out. Two other communities: Las Moras and Lapacho I, were studied and treated with anthelminthic only once due to operational issues (geographic isolation, long distance between households and lack of collaboration from public health personnel responsible for the area) that caused a poor coverage in the first intervention and prevented subsequent interventions. In two communities: Tapiete and Pablo Secretario, a first school intervention was made and no successive deworming was applied because the baseline prevalence was considered too low (< 20%) to require regular PC. Baseline assessment was performed taking into consideration the results of the six communities, while for the longitudinal analysis only the two communities (n = 2685) where baseline and follow up surveillance were performed were included. Fig 1 shows the flow diagram of the enrollment of participants in the study.

### Baseline assessment

We found a heterogeneous distribution of STH prevalence between communities varying from 11% to 72%, urban communities (Pablo Secretario and Tapiete) had a significantly lower prevalence than peri-urban communities (Lapacho Alto, Kilometro 6, Lapacho I and Las Moras), p<0.001. Table 1 details the baseline prevalence of STH infections and the coverage reached with the first intervention in each one of the studied communities.

**Table 1:** Description of the baseline STH prevalence and coverage of the first Preventive Chemotherapy (PC) campaign by community.

Community	Soil-transmitted helminth prevalence			First Preventive Chemotherapy coverage		
	N	%	95% IC	N	%	95% CI
Kilometro 6	75/130	57.7%	49–66	1153/1435	80.3%	78–82
Lapacho I	21/34	61.8%	44–79	370/572	64.6%	60–68
Lapacho Alto	48/67	71.6%	60–83	505/547	92.3%	89–94
Las Moras	23/38	60.5%	44–77	351/873	40.2%	36–43
Pablo Secretario	17/81	21.0%	11–30	621/672	92.4%	90–94
Tapiete	5/47	10.6%	4–23	778/792	98.2%	97–99
**Total**	**189/397**	**47.6%**	**43–53**	**3778/4891**	**77%**	**76–78**

Among the 6 communities, hookworm (34%) and *Strongyloides stercoralis* (26%) were the most frequent parasitological findings. Regarding hookworm, species identification was done through Harada-Mori in 73 of the 135 positive cases: 63 were *A*. *duodenale*; 9 were *N*. *americanus* and 1 was a co-infection of both species. The other 62 cases were negative in Harada-Mori. Therefore, *A*. *duodenale* accounted for 86% of the hookworm cases identified by species. A total of 104 *S*. *stercoralis* cases were diagnosed, 53 of them were detected by parasitological exam, 53 were detected by NIE-ELISA serology and 18 were positive by both methods. Table 2 summarizes a baseline description of the study population and the findings in the survey group.

**Table 2:** Baseline characteristics of the study population.

Characteristic	N	%	95% CI
**Median age ± IQR** Age group* - Preschool age children (PSAC) (0 to 4 years-old) - School age children (SAC) (5 to 14 years-old) - Adolescents and adults (≥15 years-old) - Without data	19·2 ± 22 540/5070 1603/5070 1726/5070 1201/5070	14% 41.4% 44.6%	13–15 40–43 43–46
**Female sex**	2504/5070	49.4%	48–51
Community* Kilometro 6 Lapacho I Lapacho Alto Las Moras Pablo Secretario Tapiete Without data	1784/5070 648/5070 937/5070 751/5070 125/5070 707/5070 118/5070	35.2% 12.8% 18.5% 14.8% 2.5% 13.9%	34–36 12–14 17–19 14–16 2–3 13–15
**Drinking water source** Improved Unimproved	4860/5070 55/5070	98.9% 1.1%	98–99 0.8–1.4
**Sanitation** Improved Unimproved Without data	898/5070 4003/5070 169/5070	18.3% 81.7%	17–19 80–83
**STH infection prevalence** Any STH **#** Hookworm *S*. *stercoralis* **#** *A*. *lumbricoides* *T*. *trichiura*	189/397 135/397 104/397 15/397 3/397	47.6% 34% 26.2% 3.7% 0.8%	42–53 29–39 22–31 2–6 0.1–2
Children´s nutritional status* Stunting§ Underweight¥ Wasting¶ **Mean z-scores ± SD** Mean height for age z-score (HAZ) ± SD (n) Mean weight for age z-score (WAZ) ± SD (n) Mean weight for height z-score (WHZ) ± SD (n)	157/898 26/625 4/244 -0.86 ± 1.·2 (898) -0.21 ± 1.·08 (625) 0.80 ± 1.·08 (244)	17.5% 4.2% 1.6%	15–20 2–6 0.4–4
**Anemia** **Mean hemoglobin ± SD by age (reference value)** - Preschool age children (PSAC) - School age children (SAC) - Female adolescents and adults - Male adolescents and adults	127/409 9.9 ± 1.6 (11.·5 gr/dl) 12.·5 ± 1.4 (12 gr/dl) 11.·2 ± 1.·4 (12 gr/dl) 13.1 ± 1.5 (13 gr/dl)	31.1%	26–36

# Prevalence estimated including patients with positive results in the stool exam and/or positive NIE-ELISA test for *S*. *stercoralis*.

* WHZ is a parameter calculated only for children below 5 years old. §Stunting (HAZ <-2 SD from the international reference median value)

¥Underweight (WAZ <-2 SD from the international reference median value) or

¶ Wasting (WHZ <-2 SD from the international reference median value)

We found significant differences in mean hemoglobin level, median eosinophil count and mean HAZ according to STH infection status at the individual level (uninfected versus infected subjects). No significant differences were found in mean WAZ for children aged 1 to 15 years old and WHZ of children aged 1 to 4 years old. Table 3 displays the comparison of hematological and nutritional results between infected and uninfected groups.

**Table 3:** Comparison of baseline measurements between subjects of the surveillance group infected and uninfected with hookworm and S. stercoralis (n = 397).

Measurement	Hookworm infection		P value	*S*. *stercoralis* infection		P value
	Uninfected	Infected		Uninfected	Infected	
Hemoglobin (mean ±SD)	12.5±1.7 (n = 117)	11.2 ±1.8 (n = 66)	< 0.001	12.3±1.7 (n = 109)	11.6±1.8 (n = 74)	0.008
Eosinophil count (median ± IQR)	807±1061 (n = 117)	1882±1194 (n = 66)	< 0.001	985±1060 (n = 109)	1503±1192 (n = 74)	0.003
Height for age z-score (mean ±SD)	-0.68±1.2 (n = 110)	-1.12±1.2 (n = 78)	0.014	-0,79±1.2 (n = 129)	-1.03±1.2 (n = 59)	0.205
Weight for age z-score(mean ±SD)	-0.22±1.1 (n = 75)	-0.42±1.1 (n = 59)	0.29	-0.39±1.1 (n = 93)	-0.14±1.1 (n = 41)	0.229
Weight for height z-score(mean ±SD)	0.86±1.1 (n = 28)	0.49±1.1 (n = 17)	0.28	0.47±1.1 (n = 31)	1.27±1.1 (n = 14)	0.032

**Fig 2:**
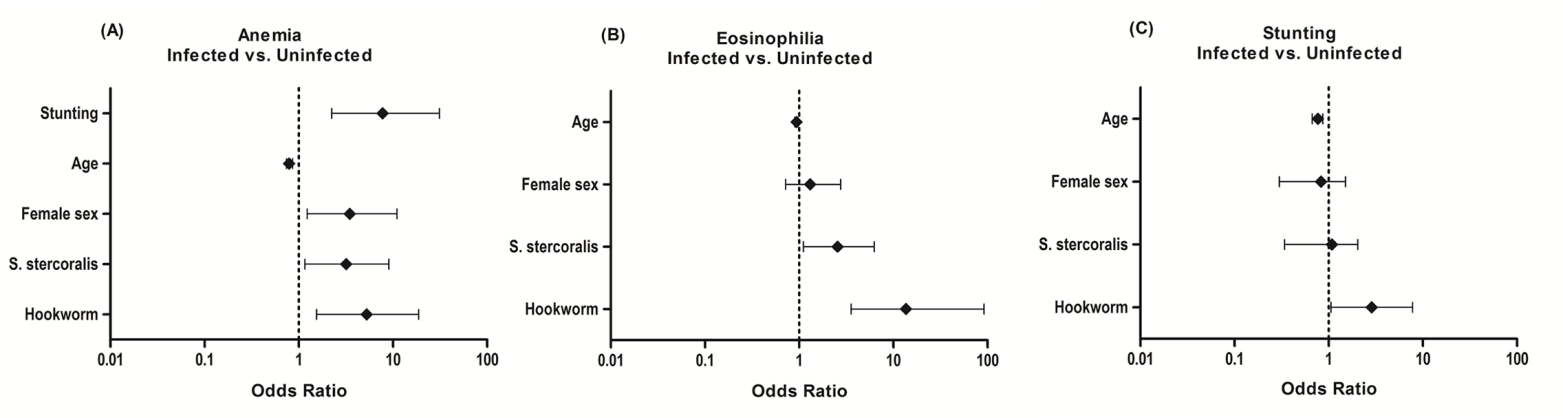
Adjusted ODDS Ratios and 95% Confidence Intervals of the associations of hookworm infection and *S*. *stercoralis* infection with a) anemia; b) eosinophilia, and c) stunting.

Hookworm infection was significantly associated with anemia (odds ratio [OR] = 5.5; 95% CI: 2.7–11.4); eosinophilia (OR = 7.48; 95% CI: 3.0–20.8) and stunting (OR = 1.7; 95% CI: 0.8–3.8). *S*. *stercoralis* infection was also significantly associated with anemia (OR = 3.5; 95% CI: 1.7–7.1) and eosinophilia (OR = 2.3; 95% CI: 1.2–4.9) but not with stunting (OR = 1.01; 95% CI: 0.4–2.3). Fig 2 displays the adjusted odds ratios after controlling for potential confounders through multivariate logistic regression models.

**Fig 3:**
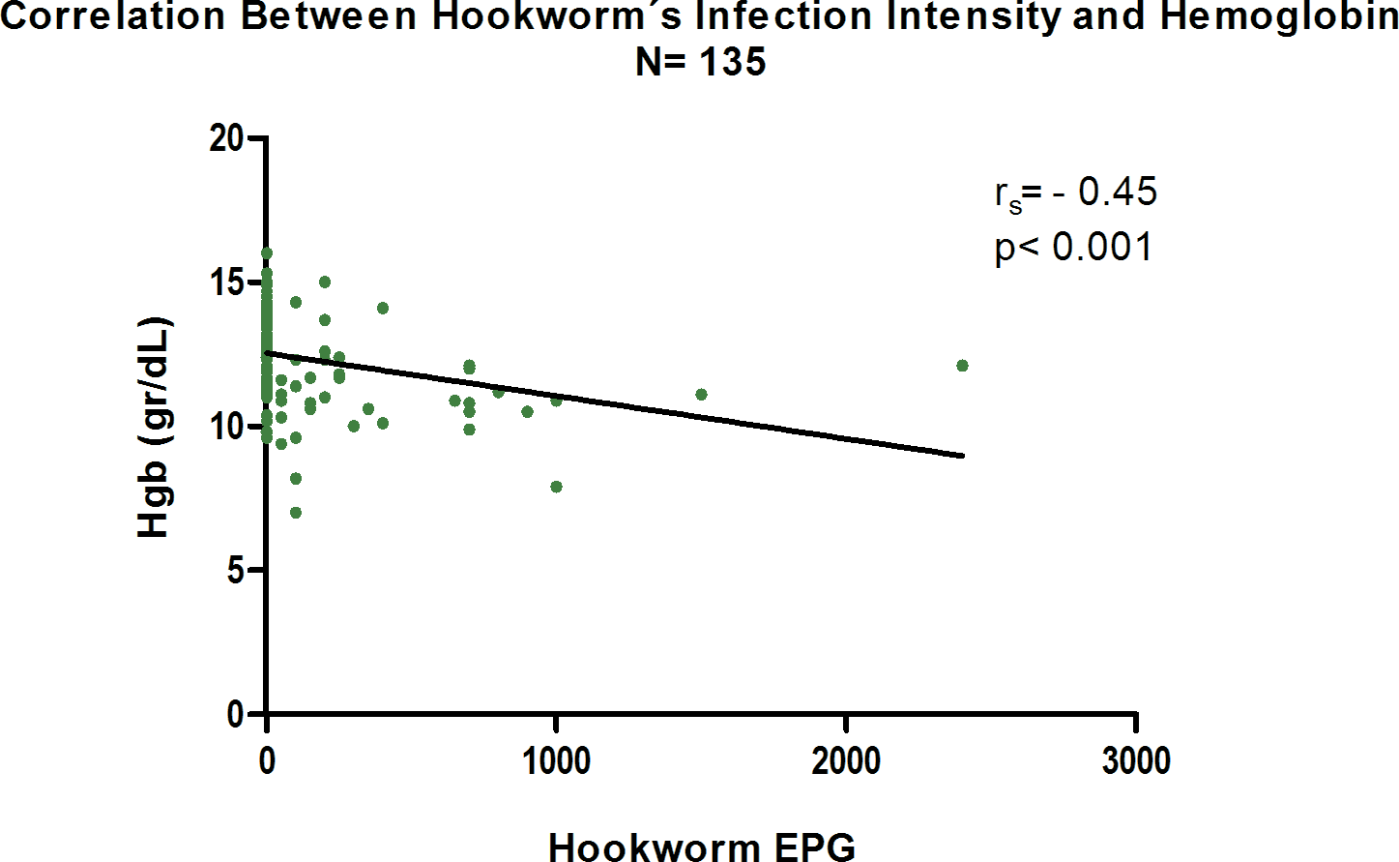
Correlation between hookworm´s infection intensity and hemoglobin level.

We found a linear negative correlation between hookworm intensity, calculated as EPG and hemoglobin level. Non-parametric Spearman´s coefficient of correlation (r_s_) = -0.46; p< 0.001. Fig 3 shows the scatter plot of this correlation. It should be noted that the frequency of heavy and moderate intensity infections was low with most cases having light infections. However, even light egg burden was significantly associated with anemia since 65% (95% CI: 48–81) of the subjects with light hookworm infection were anemic compared to 12% (95% CI: 5–19) of uninfected subjects.

### Longitudinal assessment

Lapacho Alto and Kilometro 6 inhabitants (n = 2685) received three community deworming interventions. We found significant improvements in the prevalence of STH infection, anemia, eosinophilia and children´s chronic malnutrition (stunting) after community-based PC with albendazole and ivermectin. Table 4 summarizes the indicators measured at baseline (before first PC) and at follow-up (before second and third interventions).

**Table 4:** Comparison of prevalence before and after PC in Lapacho Alto and Kilometro 6 communities.

Measure	Baseline (B) Proportion; % (95% CI)	1st Follow up (1FU) Proportion; % (95% CI)	2nd Follow up (2FU) Proportion; % (95% CI)	p1 (B-1FU) p2 (1FU-2FU) p3 (B-2FU)
**STH prevalence (Stool exam +NIE-ELISA)**	123/197; 62·4% (55–69)	30/130; 23·1% (15–31)	29/181; 16% (10–22)	p1 <0·001 p2 = 0·156 p3 <0·001
***S*. *stercoralis* prevalence** **(Stool exam)**	33/197; 16·8% (11–22)	10/130; 7·7% (3–12)	6/181; 3·3% (0.4–6)	p1 = 0·027 p2 = 0·143 p3 <0·001
***S*. *stercoralis* prevalence** **(NIE-ELISA)**	76/149; 51% (42–59)	86/153; 56·2% (48–64)	16/117; 13·7% (7–20)	p1 = 0·428 p2<0·001 p3<0·001
**Hookworm prevalence**	93/197; 47·2% (40–54)	15/130; 11·5% (6–17)	20/181; 11% (6–16)	p1<0·001 p2 = 0·962 p3<0·001
**Anemia**	84/151; 55·6% (47–64)	17/156; 10·9% (6–16)	24/165; 14·5% (9–20)	p1<0·001 p2 = 0·417 p3<0·001
**Eosinophilia**	110/150; 73·3% (66–81)	67/156; 42·9% (35–51)	50/152; 32·9% (25–41)	p1<0·001 p2 = 0·089 p3<0·001
**Stunting§**	118/613; 19·2% (16–22)	68/428; 15·9% (12–19)	46/368; 12·5% (9–16)	p1 = 0·189 p2 = 0·208 p3 = 0·009
**Underweight¥**	16/456; 3·5% (2–5)	6/340; 1·8% (0.2–3)	12/297; 4% (2–6)	p1 = 0·205 p2 = 0·136 p3 = 0·857
**Wasting¶**	4/198; 2% (0.5–5)	5/131; 3·8% (1–9)	4/113; 3·5% (1–9)	p1 = 0·526 p2 = 0·810 p3 = 0·649

§Stunting (HAZ <-2 SD from the international reference median value)

¥Underweight (WAZ <-2 SD from the international reference median value) or

¶ Wasting (WHZ <-2 SD from the international reference median value)

**Fig 4:**
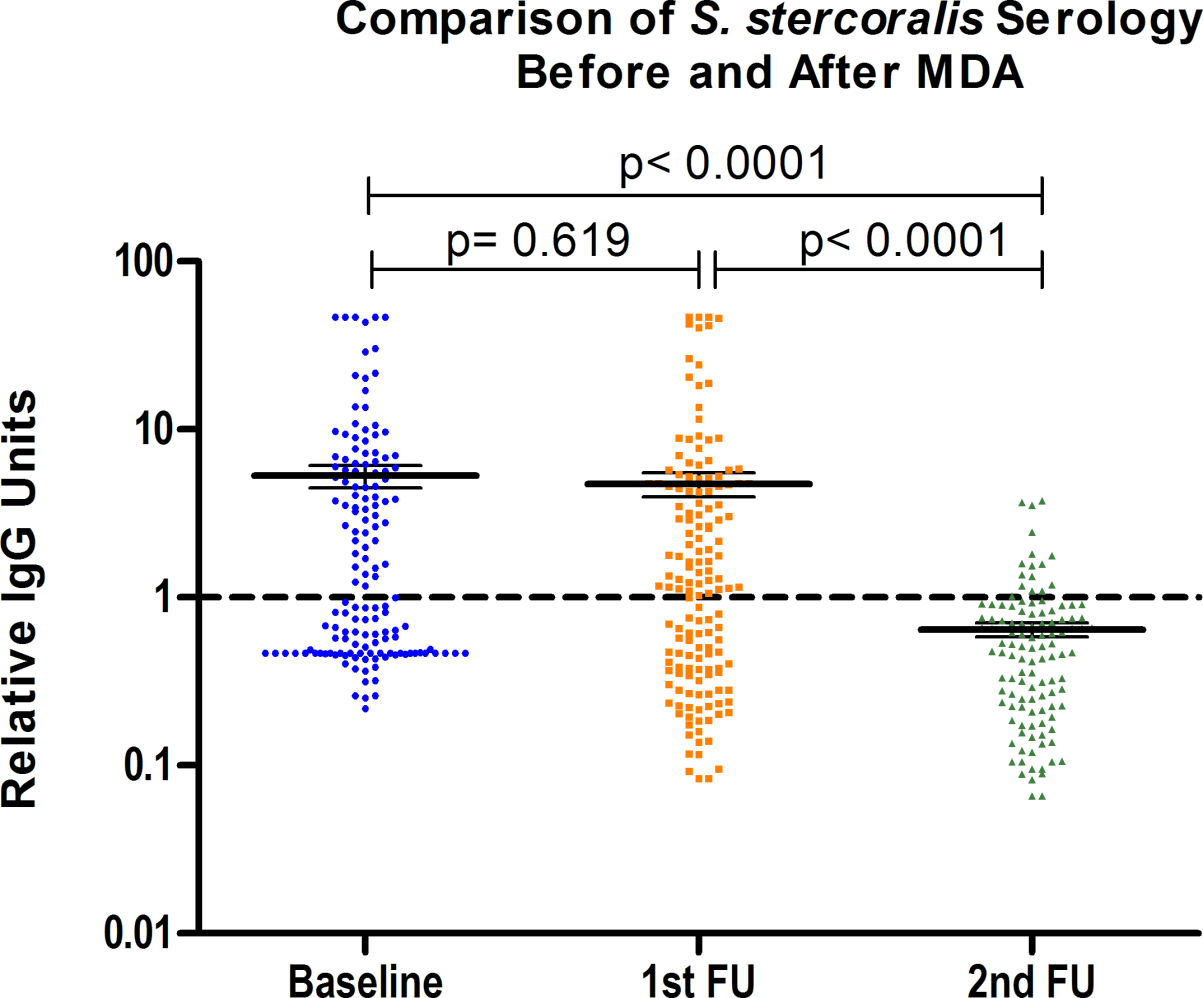
Comparison of relative IgG titers against *S*. *stercoralis*, between baseline and first and second follow-up*. * Relative IgG titers (measured IgG units/selected cutoff) reported.

We found a significant decrease in the cumulative prevalence of STH infection and in the species-specific prevalence of hookworm and *S*. *stercoralis* after the first intervention. Hookworm infection intensity decreased with deworming, showing fewer moderate and no heavy intensity infections at follow-up. We were not able to calculate hookworm’s Eggs Reduction Rate (ERR) after deworming for several reasons that attempted against the validity of this indicator. The number of stool samples collected at baseline and follow up was below the recommended sample size of 200 for this type of calculation[32]. Follow up infection intensity was assessed much later than the recommended two weeks after PC; therefore, reinfection could have occurred between baseline and follow-up assessments. Not all the samples that tested positive for hookworm through other techniques showed eggs to be counted through McMaster method, suggesting that those cases correspond to low-intensity infections. [32]. *S*. *stercoralis*´s seroprevalence showed non-significant changes after the first intervention but diminished significantly after the second intervention. Fig 4 shows the evolution of relative IgG titers across the study.

**Fig 5:**
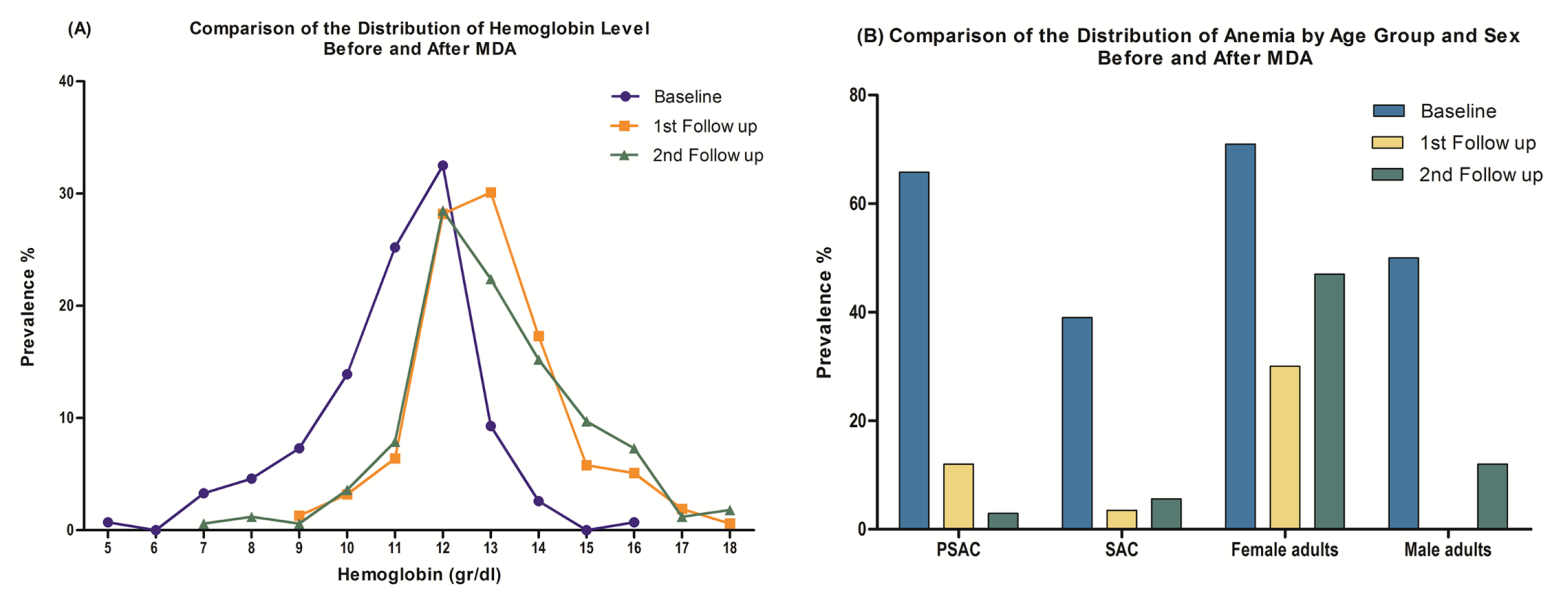
Hematological indicators along the study in Lapacho Alto and Kilometro 6 communities; A) evolution of hemoglobin level after first and second deworming and B) changes in prevalence of anemia by age and gender.

Anemia and eosinophilia prevalence and severity showed a significant response to the first intervention. When comparing hemoglobin levels between baseline and follow up surveys we found that mean hemoglobin level increased with deworming but also the curve shifted to the right showing higher minimum and maximum levels and eliminating the most extreme cases of anemia. Age and gender distribution of anemia also showed a modification following deworming. At baseline the most affected groups were PSAC and adult women but SAC and adult men were affected as well. In follow-up surveys the prevalence in PSAC, SAC and adult men declined to low levels, while adult women remained anemic; although with lower prevalence (Fig 5).

**Fig 6:**
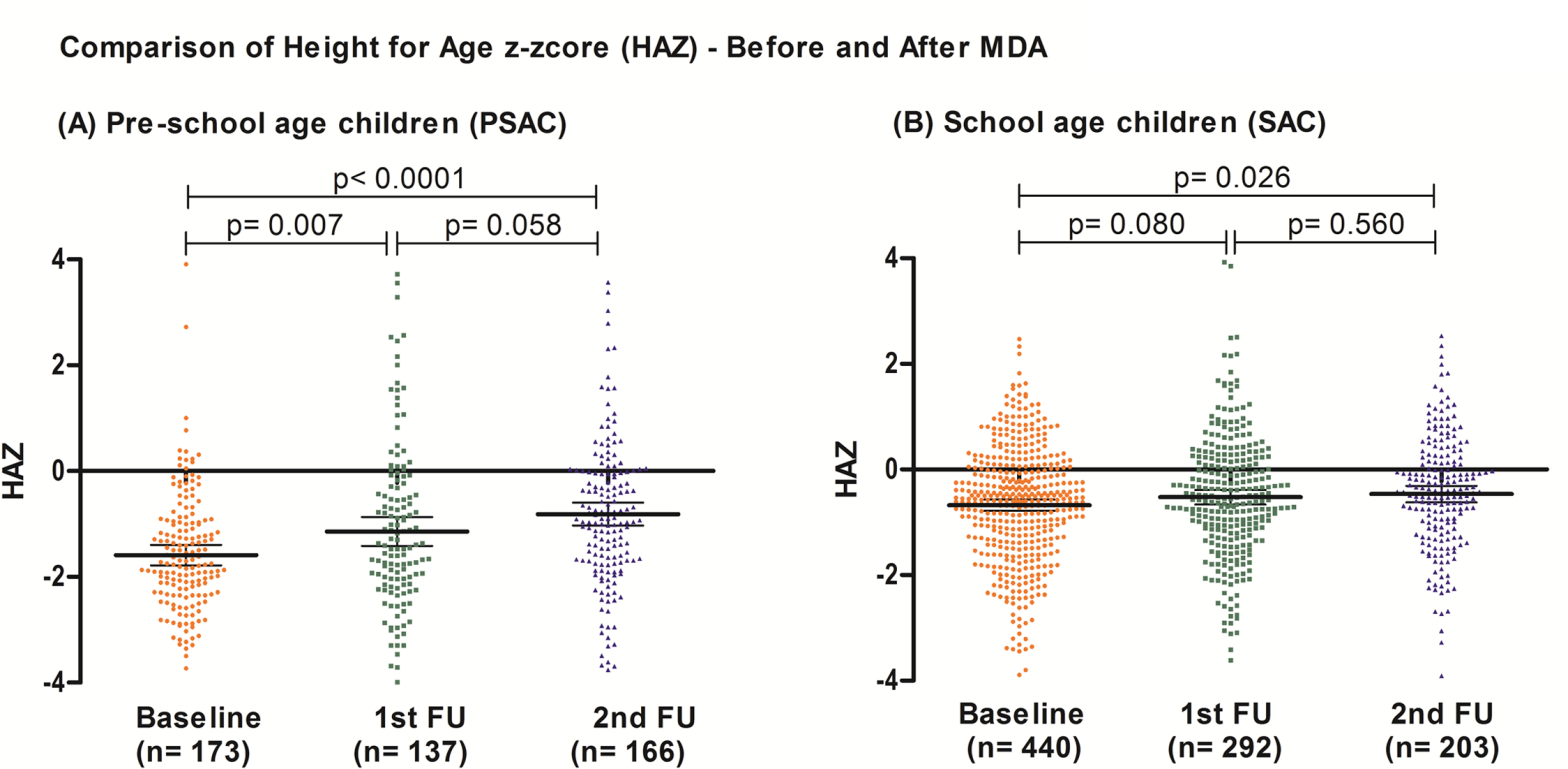
Comparison of mean height for age z-score (HAZ) between baseline and follow up by age group (A) pre-school age children (PSAC and (B) school age children.

We found an impact of deworming on stunting prevalence after two deworming campaigns but no changes in underweight and wasting, which were infrequent already at baseline. The severity of the deficit in height showed improvement after one PC intervention: baseline mean HAZ = -0.93 (SD = 1.2) was statistically different than the measurement at first follow up which had a mean HAZ = -0.72 (SD = 1.3) (p = 0.009); although it should be noted that mean HAZ remained negative after PC. When discriminating the evolution of HAZ in PSAC from SAC, we found a significant difference in PSAC between baseline and first follow up and between first and second follow up. While SAC, showed no difference in HAZ after deworming. However, the number of HAZ observations within each group was below the minimum of 400 recommended to perform comparisons. Fig 6 shows the evolution of HAZ values in PSAC and SAC.

## Discussion

The current WHO strategy for the control of STH as a public health problem relies on the use of PC with albendazole or mebendazole for those groups and countries that carry the heaviest burden of disease. The long term and sustainable solution for the STH problem probably remains more closely linked to the provision of water and sanitation. Such an approach will impact several NTDs while addressing broader goals of shared prosperity and equity [33]. While aiming for coverage indicators to achieve public health and morbidity goals, evidence supporting the relationship between coverage and morbidity in STH is still incomplete. With the understanding that WHO recommendations are a minimum set of goals for resource limited settings, this report describes a strategy for STH control in resource-limited communities with significant deficits in sanitation, where a primary care public health system is in place and *Strongyloides stercoralis* has been described at high prevalence [23,28], through a comprehensive baseline survey and a community based intervention with albendazole-ivermectin, achieving significant impact on morbidity indicators.

The significance of STH as a public health problem in the study region was confirmed in this study both in terms of prevalence and morbidity indicators [22,28], with a baseline prevalence of 48% (IC95% 42–53%). Most of the prevalence was due to hookworm and *S*. *stercoralis*. We believe that the scarcity of *A*. *lumbricoides* and *T*. *trichiura* infections in our study population might be due to the wide distribution of piped water in the households, which might have a protecting effect against orally acquired infections. Similarly, the high prevalence of hookworm and *S*. *stercoralis* found might be related to the lack of sanitation facilities in most households, since this could increase the risk of infection by skin-penetrating species. This selectiveness of the risk factors (unimproved sanitation and unimproved water access) to the route of transmission of each STH species has been explored in a previous study of our group [23]. An alternative explanation for the low frequency of *A*. *lumbricoides* and *T*. *trichiura* infection could be the issue that we used only two methods for the detection of these species, compared to the three or five methods used for the diagnosis of hookworm and *S*.*stercoralis* respectively. However, the sensitivity of the two methods that detected eggs (sedimentation/concentration and McMaster) is appropriate (around 90%) as reported in other studies [34,35]. As a matter of fact, the use of Harada-Mori did not add new diagnosis of hookworm to the findings of concentration, this method only contributed with the specie identification; while additional cases of *S*. *stercoralis*, that went unnoticed with concentration, were detected through Baermann or agar plate cultures, suggesting an adequate sensitivity of sedimentation/concentration for the detection of eggs but a limited capacity to find larvae.

The prevalence of anemia of 31% puts these communities in the category of “Severe public health significance” according to WHO definitions [12]. Anemia was significantly associated with hookworm and *S*. *stercoralis* infection in the baseline cross-sectional analysis. Even though the design of the study is not proper to demonstrate causality, the existence of previous evidence linking STH infections (especially hookworm) with anemia, explained by already well known pathogenic mechanisms [13,36–38]; added to the fact that hematologic parameters improved in the population after the intervention with anthelminthic (and nothing else) make us infer a probably causal relationship between infection and anemia. Guidelines for anemia and STH both recommend deworming as a significant although not sufficient measure to improve the public health situation of communities with high prevalence of anemia[11,12,39].

Impact indicators following PC demonstrated significant reductions in terms of prevalence and morbidity. Statistically significant changes in STH and anemia prevalence were both achieved after a single intervention. Interestingly, the residual anemia in the post intervention assessment was concentrated in adult females, the group that has a significant complementary cause of anemia and depleted iron stores due to menstrual blood loses; still, this group also benefited from the intervention lowering the prevalence of anemia. These results highlight the vulnerability of this group and the need for STH control measures that target women of childbearing age [40,41]. In accordance with other studies showing intervals of up to a year, *S*. *stercoralis* prevalence measured through NIE-ELISA only showed significant decreases in prevalence after 2 rounds of MDA [42].

The use of a comprehensive diagnostic panel allowed the identification of *S*. *stercoralis* as a significant pathogen and its morbidity, justifying the choice of the selected drug regimen. Should the diagnostic approach have been limited to a quantitative egg detection method, like Kato-Katz, McMaster`s or Mini-FLOTAC, this species would have gone completely unnoticed. Another significant observation from this diagnostic approach is the recognition that low burden hookworm infections carry a significant burden of anemia (Fig 3), which is different than in previous reports [43]. If confirmed, these results should stimulate a reassessment of the current approach that links morbidity solely to moderate and high burden infections. This finding is possibly related to co-existing conditions present in these communities, such as inadequate iron dietary intake and depleted iron stores of the subjects, which increases the likelihood that even relatively small chronic blood losses cause anemia; therefore, our results might not be generalizable.

The impact on growth indicators in children, which are probably multifactorial, showed that height-for-age z-scores (HAZ) were significantly lower in STH infected children at baseline; moreover, as indicated by WHO nutritional guidelines [27], the fact that the mean HAZ is below zero suggests that the whole population is affected or at risk. Stunting, as the nutritional parameter indicating more severe compromise of HAZ, was significantly associated with hookworm infection and underwent significant improvement after the second (but not the first) drug intervention, possibly indicating the interval of time free of STH needed to observe changes in this growth parameter. Even though the mean HAZ significantly increased after two PC interventions it remained negative suggesting that a chronic nutritional impairment persisted despite deworming; these might be related to the relative short time of follow up or to concomitant causes of malnutrition such as deficient dietary intake. We were not able to demonstrate any association of hookworm or *S*. *stercoralis* infection with weight for age z-score (WAZ) and weight for height z-score (WHZ), or impact of deworming on these parameters.

The association of STH infection with anemia and nutritional impairment needs to be considered in the context of possible confounding factors such as family income. Poverty is a well-known risk factor for STH infection but may also be a cause or contributing cause of anemia and malnutrition, since poor families tend to have diets insufficient in quantity and quality with low iron content. We did not include poverty in the multivariate analysis to confirm or discard a confounding effect in the association found between hookworm or *S*. *stercoralis* infection and anemia or malnutrition because data of family income was unavailable. However, the homogeneity of the socio-economic status of the study population makes us infer that the low family income was similarly distributed in the infected and uninfected groups.

Our study has limitations to be considered: first, the surveillance samples at baseline and follow-up are not matched samples; therefore, we compared the results before and after PC by community and not individually. In a recently published article, we demonstrated in a smaller group in a community project close to the study area, with paired samples, that after a year of follow-up, decreases in NIE-ELISA antibody titers were similar to those observed in this study [44]. Second, some communities that were studied at baseline and treated for the first time were not surveyed at follow up and did not received successive deworming; we included these communities in a cross sectional baseline assessment looking for associations of STH infection but for the prospective analysis only the communities where follow up surveillance was performed were compared. Third, false negative results could occur due to suboptimal sensitivity of the diagnostic methods, which can overestimate the impact of deworming since a reduction in burden might be read and interpreted as a negative result. To deal with this limitation, various diagnostic methods were used. More sensitive methods such as PCR for the detection of STH might overcome this limitation [45]. Fourth, some self-selection bias might have happened related to the fact that some individuals selected for surveillance refused to participate and those among the selected that provided samples for the study might be at higher risk of infection. Fifth, the coverage reached with the interventions was not optimal, which could underestimate the real effectiveness of the drug regimen. Finally, we do not report the impact of deworming on infection intensity; due to the limitations of our study for the estimation of ERR, even at group level. Most of our limitations are typical of population-based trials in a resource-restricted setting. Despite these limitations, the analysis performed, including multivariate analysis, and the strength of the associations and effects, in the context of no other changes in conditions than the PC, provide the foundations for our interpretations regarding the benefits of PC with albendazole and ivermectin.

As previously mentioned, we found difficulty in conducting PC in some communities where the collaboration of the sanitary agents responsible for the areas was meager and were geographically isolated which resulted in low coverage rates and the discontinuation of the study in those communities. For those situations, a school based intervention, as recommended by WHO [46], is a more feasible option, even though with more limited results in terms of morbidity reduction since women and preschool age children are not targeted.

The drug regimen used in these interventions has been used in a few clinical trials for the treatment of STH, mostly aiming at improving efficacy for *T*. *trichiura* [20,47], however most of the data regarding its use, safety and even impact on STH, comes from lymphatic filariasis (LF) programmes in areas with onchocerciasis, where millions of individuals have been treated, highlighting the safety of this regimen [48,49]. Since currently most PC programs are dependent on drug donations, the addition of IVM needs to be considered in the context of the critical issue of drug availability.

This is, to our knowledge, the first report of an intervention of PC with albendazole-ivermectin for pure STH control; its positive short-term effects on morbidity are echoed by recent reports proposing this drug combination as a feasible strategy for delaying the emergence of resistance and offering improved efficacy against *T*. *trichiura* and *S*. *stercoralis* in clinical trials and mathematical models [20,50–52]. In summary, albendazole/ivermectin applied as community based PC in communities with high prevalence of hookworm and *S*. *stercoralis* resulted in significant reduction of STH prevalence and improvements in anemia in the general population and nutritional status in children. These results should be further explored in randomized-controlled trials with cost-effectiveness evaluation to overcome the limitations of our study.

## Supporting information

S1 Checklist STROBE statement for observational studies checklist. (PDF)

S1 Text ReadMe information for the dataset Tartagal Argentina 5070. (DOCX)

S1 Dataset TARTAGAL Argentina 5070. (CSV)

## References

[1] World Health Organization. Soil Transmitted Helminthiases. Eliminating soil-transmitted helminthiases as a public health problem in children Progress report 2001–2010 and strategic plan 2011–2020. Geneva, Switzerland: WHO Press; 2012.

[2] Bethony J, Brooker S, Albonico M, Geiger SM, Loukas A, Diemert D, et al Soil-transmitted helminth infections: ascariasis, trichuriasis, and hookworm. Lancet. 2006;367: 1521–32. doi: 10.1016/S0140-6736(06)68653-4 16679166 10.1016/S0140-6736(06)68653-4

[3] World Health Organization. Working to overcome the global impact of neglected tropical diseases First WHO report on neglected tropical diseases. Crompton DWT, editor. France: WHO Press; 2010.

[4] Olsen A, van Lieshout L, Marti H, Polderman T, Polman K, Steinmann P, et al Strongyloidiasis—the most neglected of the neglected tropical diseases? Trans R Soc Trop Med Hyg. 2009;103: 967–72. doi: 10.1016/j.trstmh.2009.02.013 19328508 10.1016/j.trstmh.2009.02.013

[5] Krolewiecki AJ, Lammie P, Jacobson J, Gabrielli AF, Levecke B, Socias E, et al A Public Health Response against Strongyloides stercoralis: Time to Look at Soil-Transmitted Helminthiasis in Full. PLoS Negl Trop Dis. 2013;7: 1–7. doi: 10.1371/journal.pntd.0002165 23675541 10.1371/journal.pntd.0002165 PMC3649958

[6] Hotez PJ, Bundy D a P, Beegle K, Brooker S, Drake L, De Silva N, et al Helminth Infections: Soil-Transmitted Helminth Infections and Schistosomiasis In: Jamison DT, Breman JG, Measham AR, Alleyne G, Claeson M, Evans DB, et al., editors. Disease Control Priorities in Developing Countries. Second edi. Washington (DC): Oxford University Press and The World Bank; 2006 pp. 467–482. doi: 10.1596/978-0-821-36179-5/Chpt-24

[7] Watkins WE, Pollitt E. “Stupidity or worms”: do intestinal worms impair mental performance? Psychol Bull. 1997;121: 171–91. doi: 10.1037/0033-2909.121.2.171 9100486 10.1037/0033-2909.121.2.171

[8] Nokes C, Grantham-McGregor SM, Sawyer a W, Cooper ES, Bundy D a. Parasitic helminth infection and cognitive function in school children. Proc Biol Sci. 1992;247: 77–81. doi: 10.1098/rspb.1992.0011 1349184 10.1098/rspb.1992.0011

[9] Stoltzfus RJ, Kvalsvig JD, Chwaya HM, Montresor a., Albonico M, Tielsch JM, et al Effects of iron supplementation and anthelmintic treatment on motor and language development of preschool children in Zanzibar: double blind, placebo controlled study. Bmj. 2001;323: 1389–1389. doi: 10.1136/bmj.323.7326.1389 11744561 10.1136/bmj.323.7326.1389 PMC60982

[10] Crompton DWT, Nesheim MC. Nutritional impact of intestinal helminthiasis during the human life cycle. Annu Rev Nutr. 2002;22: 35–59. doi: 10.1146/annurev.nutr.22.120501.134539 12055337 10.1146/annurev.nutr.22.120501.134539

[11] Shaw JG, Friedman JF. Iron deficiency anemia: focus on infectious diseases in lesser developed countries. Anemia. 2011;2011: 260380 doi: 10.1155/2011/260380 21738863 10.1155/2011/260380 PMC3124144

[12] Stoltzfus RJ, Mullany L, Black RE. Iron deficiency anaemia. Assesment, Prevention and Control A guide for programme managers. Hempstead R, editor. WHO Report Geneva, Switzerland; 2004.

[13] Lwambo NJ, Bundy DAP, Medley GF. A new approach to morbidity risk assessment in hookworm endemic communities. Epidemiol Infect. 1992;108: 469–81. 1601081 10.1017/s0950268800049980 PMC2272209

[14] Brooker S, Clements a. C a, Bundy D a P. Global Epidemiology, Ecology and Control of Soil-Transmitted Helminth Infections. Adv Parasitol. 2006;62: 221–261. doi: 10.1016/S0065-308X(05)62007-6 16647972 10.1016/S0065-308X(05)62007-6 PMC1976253

[15] WHO. Preventive chemotherapy in human helminthiasis: coordinated use of anthelminthic drugs in control interventions: a manual for health professionals and programme managers Crompton DWT, editor. WHO Library. Geneva, Switzerland: WHO Press; 2006.

[16] Ahmed A, Al-Mekhlafi HM, Al-Adhroey AH, Ithoi I, Abdulsalam AM, Surin J. The nutritional impacts of soil-transmitted helminths infections among Orang Asli schoolchildren in rural Malaysia. Parasit Vectors. 2012;5: 119 doi: 10.1186/1756-3305-5-119 22704549 10.1186/1756-3305-5-119 PMC3419660

[17] Humphries D, Simms BT, Davey D, Otchere J, Quagraine J, Terryah S, et al Hookworm infection among school age children in Kintampo north municipality, Ghana: nutritional risk factors and response to albendazole treatment. Am J Trop Med Hyg. 2013;89: 540–8. doi: 10.4269/ajtmh.12-0605 23836564 10.4269/ajtmh.12-0605 PMC3771297

[18] Albonico M, Allen H, Chitsulo L, Engels D, Gabrielli A-F, Savioli L. Controlling soil-transmitted helminthiasis in pre-school-age children through preventive chemotherapy. PLoS Negl Trop Dis. 2008;2: e126 doi: 10.1371/journal.pntd.0000126 18365031 10.1371/journal.pntd.0000126 PMC2274864

[19] Anselmi M, Buonfrate D, Guevara Espinoza A, Prandi R, Marquez M, Gobbo M, et al Mass Administration of Ivermectin for the Elimination of Onchocerciasis Significantly Reduced and Maintained Low the Prevalence of Strongyloides stercoralis in Esmeraldas, Ecuador. PLoS Negl Trop Dis. 2015;9: e0004150 doi: 10.1371/journal.pntd.0004150 26540412 10.1371/journal.pntd.0004150 PMC4635009

[20] Speich B, Ali SM, Ame SM, Bogoch II, Alles R, Huwyler J, et al Efficacy and safety of albendazole plus ivermectin, albendazole plus mebendazole, albendazole plus oxantel pamoate, and mebendazole alone against Trichuris trichiura and concomitant soil-transmitted helminth infections: a four-arm, randomised controlled t. Lancet Infect Dis. 2015;15: 277–284. doi: 10.1016/S1473-3099(14)71050-3 25589326 10.1016/S1473-3099(14)71050-3

[21] Romani L, Whitfeld MJ, Koroivueta J, Kama M, Wand H, Tikoduadua L, et al Mass Drug Administration for Scabies Control in a Population with Endemic Disease. N Engl J Med. 2015;373: 2305–2313. doi: 10.1056/NEJMoa1500987 26650152 10.1056/NEJMoa1500987

[22] Taranto NJ, Cajal SP, De Marzi MC, Fernández MM, Frank FM, Brú a M, et al Clinical status and parasitic infection in a Wichí Aboriginal community in Salta, Argentina. Trans R Soc Trop Med Hyg. 2003;97: 554–8. 15307425 10.1016/s0035-9203(03)80026-3

[23] Echazú A, Bonanno D, Juarez M, Cajal SP, Heredia V, Caropresi S, et al Effect of Poor Access to Water and Sanitation As Risk Factors for Soil-Transmitted Helminth Infection: Selectiveness by the Infective Route. PLoS Negl Trop Dis. 2015;9: 1–14. doi: 10.1371/journal.pntd.0004111 26421865 10.1371/journal.pntd.0004111 PMC4589369

[24] WHO. Worldwide prevalence of anaemia Benoist B de, McLean E, Egli I, Cogswell M, editors. WHO Report. Geneva, Switzerland: WHO Press; 2005 doi: 10.1017/S1368980008002401

[25] Kogan L, Abeya Gilardón E, Biglieri A, Mangialavori G, Calvo E, Durán P. Anemia: La desnutrición oculta Resultados de la Encuesta Nacional de Nutrición y Salud—ENNyS- 2008. Ministerio de Salud de Argentina 2008.

[26] World Health Organization and UNICEF. Joint Monitoring Programme for Water Supply and Sanitation. Core questions on drinking-water and sanitation for household surveys WHO/UNICEF Joint Monitoring Programme for Water Supply and Sanitation. WHO Press, World Health Organization, 20 Avenue Appia, 1211 Geneva 27, Switzerland; 2006.

[27] de Onis M, Blössner M. WHO Global Database on Child Growth and Malnutrition. World Health Organization, Programme of Nutrition. 1997. 10.1093/ije/dyg099 12913022

[28] Krolewiecki AJ, Ramanathan R, Fink V, McAuliffe I, Cajal SP, Won K, et al Improved diagnosis of Strongyloides stercoralis using recombinant antigen-based serologies in a community-wide study in northern Argentina. Clin Vaccine Immunol. 2010;17: 1624–1630. doi: 10.1128/CVI.00259-10 20739501 10.1128/CVI.00259-10 PMC2952987

[29] Farreras P, Rozman C. Medicina Interna—Volumen II. 16th ed Barcelona, España: ELSEVIER; 2004.

[30] Ravi V, Ramachandran S, Thompson RW, Andersen JF, Neva FA. Characterization of a recombinant immunodiagnostic antigen (NIE) from Strongyloides stercoralis L3-stage larvae. Mol Biochem Parasitol. 2002;125: 73–81. 12467975 10.1016/s0166-6851(02)00214-1

[31] Bisoffi Z, Buonfrate D, Sequi M, Mejia R, Cimino RO, Krolewiecki AJ, et al Diagnostic accuracy of five serologic tests for Strongyloides stercoralis infection. PLoS Negl Trop Dis. 2014;8: e2640 doi: 10.1371/journal.pntd.0002640 24427320 10.1371/journal.pntd.0002640 PMC3890421

[32] Vercruysse J, Albonico M, Behnke JM, Kotze AC, Prichard RK, McCarthy JS, et al Is anthelmintic resistance a concern for the control of human soil-transmitted helminths? Int J Parasitol Drugs Drug Resist. Australian Society for Parasitology; 2011;1: 14–27. doi: 10.1016/j.ijpddr.2011.09.002 24533260 10.1016/j.ijpddr.2011.09.002 PMC3913213

[33] WHO. Water sanitation and hygiene for accelerating and sustaining progress on neglected tropical diseases A global strategy 2015–2020. World Health Organization, editor. Geneva; 2015. 10.1093/inthealth/ihv073 PMC5580794 26940305

[34] Levecke B, Behnke JM, Ajjampur SSR, Albonico M, Ame SM, Charlier J, et al A comparison of the sensitivity and fecal egg counts of the McMaster egg counting and Kato-Katz thick smear methods for soil-transmitted helminths. PLoS Negl Trop Dis. 2011;5: e1201 doi: 10.1371/journal.pntd.0001201 21695104 10.1371/journal.pntd.0001201 PMC3114752

[35] Goodman D, Haji HJ, Bickle QD, Stoltzfus RJ, Tielsch JM, Ramsan M, et al A comparison of methods for detecting the eggs of Ascaris, Trichuris, and hookworm in infant stool, and the epidemiology of infection in Zanzibari infants. Am J Trop Med Hyg. 2007;76: 725–31. 17426179

[36] Stoltzfus R, Chwaya H, Tielsch J, Schulze K, Albonico M, Savioli L. Epidemiology of iron deficiency anemia in Zanzibari schoolchildren: the importance of hookworm. Am J Clin Nutr. 1997;65: 153–159. 8988928 10.1093/ajcn/65.1.153

[37] Farid Z, Patwardhan V, Darby W. Parasitism and anemia. Am J Clin Nutr. 1969;22: 498–503. 5778077 10.1093/ajcn/22.4.498

[38] Stassens P, Bergum PW, Gansemans Y, Jespers L, Laroche Y, Huang S, et al Anticoagulant repertoire of the hookworm Ancylostoma caninum. Proc Natl Acad Sci U S A. 1996;93: 2149–54. 8700900 10.1073/pnas.93.5.2149 PMC39925

[39] Stoltzfus RJ, Dreyfuss ML, Chwaya HM, Albonico M. Hookworm control as a strategy to prevent iron deficiency. Nutr Rev. 1997;55: 223–232. doi: 10.1111/j.1753-4887.1997.tb01609.x 9279058 10.1111/j.1753-4887.1997.tb01609.x

[40] Brooker S, Hotez PJ, Bundy DAP. Hookworm-Related Anaemia among Pregnant Women: A Systematic Review. 2008;2 doi: 10.1371/journal.pntd.0000291 18820740 10.1371/journal.pntd.0000291 PMC2553481

[41] Larocque R, Casapia M, Gotuzzo E, Gyorkos TW. Relationship between intensity of soil-transmitted helminth infections and anemia during pregnancy. Am J Trop Med Hyg. 2005;73: 783–789. 16222026

[42] Buonfrate D, Sequi M, Mejia R, Cimino RO, Krolewiecki AJ, Albonico M, et al Accuracy of five serologic tests for the follow up of Strongyloides stercoralis infection. PLoS Negl Trop Dis. 2015;9: e0003491 doi: 10.1371/journal.pntd.0003491 25668740 10.1371/journal.pntd.0003491 PMC4323101

[43] Kuong K, Fiorentino M, Perignon M, Chamnan C, Berger J, Sinuon M, et al Cognitive Performance and Iron Status are Negatively Associated with Hookworm Infection in Cambodian Schoolchildren. Am J Trop Med Hyg. 2016;95: 15–0813. doi: 10.4269/ajtmh.15-0813 27573634 10.4269/ajtmh.15-0813 PMC5062788

[44] Vargas P, Krolewiecki AJ, Echazú A, Juarez M, Cajal P, Gil JF, et al Serologic Monitoring of Public Health Interventions Against Strongyloides stercoralis. Am J Trop Med Hyg. The American Journal of Tropical Medicine and Hygiene; 2017; 16–0857. doi: 10.4269/ajtmh.16-0857 28719325 10.4269/ajtmh.16-0857 PMC5508900

[45] Cimino RO, Jeun R, Juarez M, Cajal PS, Vargas P, Echazú A, et al Identification of human intestinal parasites affecting an asymptomatic peri-urban Argentinian population using multi-parallel quantitative real-time polymerase chain reaction. Parasit Vectors. Parasites & Vectors; 2015;8: 380 doi: 10.1186/s13071-015-0994-z 26183074 10.1186/s13071-015-0994-z PMC4504406

[46] World Health Organization. Helminth control in school-age children. A guide for managers of control programs. Second edition. 2011;

[47] Knopp S, Mohammed KA, Speich B, Khamis IS. Efficacy of albendazole and mebendazole alone or in combination with ivermectin against Trichuris trichiura and other soil- transmitted helminths Control of helminthiases in Zanzibar. 2009; 1–12.

[48] Tisch DJ, Michael E, Kazura JW. Mass chemotherapy options to control lymphatic filariasis: A systematic review. Lancet Infect Dis. 2005;5: 514–523. doi: 10.1016/S1473-3099(05)70192-4 16048720 10.1016/S1473-3099(05)70192-4

[49] Thylefors B, Alleman MM, Twum-Danso NAY. Operational lessons from 20 years of the Mectizan Donation Program for the control of onchocerciasis. Trop Med Int Heal. 2008;13: 689–696. doi: 10.1111/j.1365-3156.2008.02049.x 18419585 10.1111/j.1365-3156.2008.02049.x

[50] Truscott JE, Turner HC, Anderson RM. What impact will the achievement of the current World Health Organisation targets for anthelmintic treatment coverage in children have on the intensity of soil transmitted helminth infections? Parasit Vectors. Parasites & Vectors; 2015;8: 551 doi: 10.1186/s13071-015-1135-4 26490544 10.1186/s13071-015-1135-4 PMC4618937

[51] Lo NC, Addiss DG, Hotez PJ, King CH, Stothard JR, Evans DS, et al Personal View A call to strengthen the global strategy against schistosomiasis and soil-transmitted helminthiasis: the time is now. 2016;3099. 10.1016/S1473-3099(16)30535-7 PMC5280090 27914852

[52] Turner HC, Truscott JE, Bettis AA, Hollingsworth TD, Brooker SJ, Anderson RM. Analysis of the population-level impact of co-administering ivermectin with albendazole or mebendazole for the control and elimination of Trichuris trichiura. Parasite Epidemiol Control. The Authors; 2016;1: 177–187. doi: 10.1016/j.parepi.2016.02.004 27430028 10.1016/j.parepi.2016.02.004 PMC4946157

