# Supplementary material for: Albendazole and ivermectin for the control of soil-transmitted helminths in an area with high prevalence of Strongyloides stercoralis and hookworm in northwestern Argentina: A community-based pragmatic study
Source: PLoS Negl Trop Dis. 2017 Oct 9;11(10):e0006003. doi: 10.1371/journal.pntd.0006003 (PMC5648268; doi:10.1371/journal.pntd.0006003)
Supplement: S1 Checklist — (PDF) [file pntd.0006003.s001.pdf]

STROBE Statement—checklist of items that should be included in reports of observational studies

Albendazole and ivermectin for the control of soil-transmitted helminths in an area with high prevalence of *Strongyloides stercoralis* and hookworm in northwestern Argentina: a community-based pragmatic study.

	Item No	Recommendation
**Title and abstract**	1	(*a*) Indicate the study's design with a commonly used term in the title or the abstract<br>Title (page 1; lines 1-3). Abstract, methodology (page 2; line 33)
		(*b*) Provide in the abstract an informative and balanced summary of what was done and what was found<br>Abstract, methodology and principal findings (pages 1-2; lines 33-46)
**Introduction**		
Background/rationale	2	Explain the scientific background and rationale for the investigation being reported<br>Introduction (pages 4-5; lines 72-112)
Objectives	3	State specific objectives, including any prespecified hypotheses<br>Introduction (pages 5-6; lines 113-122)
**Methods**		
Study design	4	Present key elements of study design early in the paper<br>Methods (page 6; lines 124-126)
Setting	5	Describe the setting, locations, and relevant dates, including periods of recruitment, exposure, follow-up, and data collection<br>Methods (page 6; line 126 and pages 6-7; lines 135-159)
Participants	6	(*a*) *Cross-sectional study*—Give the eligibility criteria, and the sources and methods of selection of participants<br>Methods (pages 7-8; lines 161-173)
Variables	7	Clearly define all outcomes, exposures, predictors, potential confounders, and effect modifiers. Give diagnostic criteria, if applicable<br>Methods (page11, lines 254-262)
Data sources/ measurement	8*	For each variable of interest, give sources of data and details of methods of assessment (measurement). Describe comparability of assessment methods if there is more than one group<br>Methods (pages 9-10; lines 193-252)
Bias	9	Describe any efforts to address potential sources of bias
Study size	10	Explain how the study size was arrived at<br>Methods (page 12; lines 264-267)
Quantitative variables	11	Explain how quantitative variables were handled in the analyses. If applicable, describe which groupings were chosen and why<br>Methods (page 12; lines 268-270)
Statistical methods	12	(*a*) Describe all statistical methods, including those used to control for confounding<br>Methods (Page 12; lines 263-281)
		(*b*) Describe any methods used to examine subgroups and interactions<br>Methods (Page 12; lines 270-275)
		(*c*) Explain how missing data were addressed

		(*d*) *Cohort study*—If applicable, explain how loss to follow-up was addressed<br>*Case-control study*—If applicable, explain how matching of cases and controls was addressed<br>*Cross-sectional study*—If applicable, describe analytical methods taking account of sampling strategy
		(*e*) Describe any sensitivity analyses
**Results**		
Participants	13*	(a) Report numbers of individuals at each stage of study—eg numbers potentially eligible, examined for eligibility, confirmed eligible, included in the study, completing follow-up, and analysed<br>Results: **Figure 1: Flow diagram of the participants and size of surveillance samples enrolled along the study** (page 13; lines 297-302)
		(b) Give reasons for non-participation at each stage<br>Results: **Figure 1: Flow diagram of the participants and size of surveillance samples enrolled along the study** (page 13; lines 297-302)
		(c) Consider use of a flow diagram<br>Results: **Figure 1: Flow diagram of the participants and size of surveillance samples enrolled along the study** (page 13; lines 297-302)
Descriptive data	14*	(a) Give characteristics of study participants (eg demographic, clinical, social) and information on exposures and potential confounders<br>Results: **Table 2: Baseline characteristics of the study population** (pages 14-15; lines 319-324)
		(b) Indicate number of participants with missing data for each variable of interest<br>Results: **Table 2: Baseline characteristics of the study population** (pages 14-15; lines 319-324)
		(c) *Cohort study*—Summarise follow-up time (eg, average and total amount)
Outcome data	15*	*Cohort study*—Report numbers of outcome events or summary measures over time
		Case-control study—Report numbers in each exposure category, or summary measures of exposure
		Cross-sectional study—Report numbers of outcome events or summary measures<br>Results: **Table 4: Comparison of prevalence before and after PC in Lapacho Alto and Kilometro 6 communities** (pages 17-18; lines 358-371)
Main results	16	(*a*) Give unadjusted estimates and, if applicable, confounder-adjusted estimates and their precision (eg, 95% confidence interval). Make clear which confounders were adjusted for and why they were included<br>Unadjusted estimates: Results (page 16; lines 334-339). Adjusted estimates: Results (page 16; lines 340-342) **Figure 2: Adjusted Odds Ratios and 95 % Confidence Intervals of the associations of hookworm infection and *S. stercoralis* infection with a) anemia; b) eosinophilia, and c) stunting**)
		(*b*) Report category boundaries when continuous variables were categorized<br>Results: **Table 2: Baseline characteristics of the study population** (pages 14-15; lines 319-324)<br>Methods (page 11; lines 244-245)
		(*c*) If relevant, consider translating estimates of relative risk into absolute risk for a meaningful time period

Other analyses	17	Report other analyses done—eg analyses of subgroups and interactions, and sensitivity analyses<br>Results (pages 15-16; lines 331-334): **Table 3: Comparison of baseline measurements between subjects of the surveillance group infected and uninfected with hookworm and S. stercoralis (n= 397)**<br>Results (pages 16; lines 350): **Figure 3: Correlation between hookworm´s infection intensity and hemoglobin level**
**Discussion**		
Key results	18	Summarise key results with reference to study objectives<br>Discussion (page 25; 541-546)
Limitations	19	Discuss limitations of the study, taking into account sources of potential bias or imprecision. Discuss both direction and magnitude of any potential bias<br>Discussion (Pages 23-24; lines 498-529)
Interpretation	20	Give a cautious overall interpretation of results considering objectives, limitations, multiplicity of analyses, results from similar studies, and other relevant evidence<br>Discussion (Pages 20-22; lines 420-497)
Generalizability	21	Discuss the generalizability (external validity) of the study results<br>Discussion (page 20; lines 470-473)
**Other information**		
Funding	22	Give the source of funding and the role of the funders for the present study and, if applicable, for the original study on which the present article is based<br>Fundación Mundo Sano provided funding for this study through an unrestricted grant, two of the co-authors (MA and SG) are members of Fundación Mundo Sano, their participation in the study was limited to study design and revision and approval of the final version of the article before submission. Funación Mundo Sano had no role in the patients recruitment; data collection, analysis and interpretation; writing, and decision to submit for publication.

*Give information separately for cases and controls in case-control studies and, if applicable, for exposed and unexposed groups in cohort and cross-sectional studies.

Note: An Explanation and Elaboration article discusses each checklist item and gives methodological background and published examples of transparent reporting. The STROBE checklist is best used in conjunction with this article (freely available on the Web sites of PLoS Medicine at http://www.plosmedicine.org/, Annals of Internal Medicine at http://www.annals.org/, and Epidemiology at http://www.epidem.com/). Information on the STROBE Initiative is available at www.strobe-statement.org.
