# Supplementary material for: Albendazole and ivermectin for the control of soil-transmitted helminths in an area with high prevalence of Strongyloides stercoralis and hookworm in northwestern Argentina: A community-based pragmatic study
Source: PLoS Negl Trop Dis. 2017 Oct 9;11(10):e0006003. doi: 10.1371/journal.pntd.0006003 (PMC5648268; doi:10.1371/journal.pntd.0006003)
Supplement: S1 Text — (DOCX) [file pntd.0006003.s002.docx]

**Read Me files for the use of the Data Base: Dataset_Tartagal_Argentina_5070.**

**Study: Albendazole and ivermectin for the control of soil-transmitted helminths prevalence and morbidity in an endemic area of northwestern Argentina: a community-based pragmatic study.**

**This data base contains the data collected throughout the study from the diagnosis methods performed and the information obtained at baseline from Forms-1, which are the forms filled in the census carried out every three months by public health personnel working in the study population.**

**Below you will find the description of the variables included in the data base; each one fills a column of the dataset.**

1. **ID:** subject identification number
2. **BirthDate:** date of birth of the subject. Code blank= missing data
3. **1_PC_Date:** date when the first preventive chemotherapy intervention with albendazole/ivermectin was performed in the community where the subject lives. Code blank= missing data.
4. **Age:** age of the study subject in years. Code blank= missing data.
5. **Age_WHO:** age of the study subject categorized in age groups according to WHO classification and coded. Code blank= missing data. Code 1= Preschool age children (0 – 4 years old). Code 2= School age children (5 – 14 years old). Code 3= Adolescents and adults (≥ 15 years old).
6. **Sex:** gender of the study subject. Code blank= missing data.
7. **Community:** site of residence of the study subject.
8. **Water_source:** source of the drinking-water available in the study household. Water supply is considered improved if drinking-water source is a) piped household water; b) public taps or standpipes; c) protected dug wells; d) protected springs; or e) tube wells or boreholes. While the use of surface water or drinking-water from unimproved sources (unprotected dug well, bottled water, unprotected springs or cart with small tank/drum) is considered unimproved water supply. As described in the sanitation and drinking-water ladders of the WHO/UNICEF Joint Monitoring Programme (JMP) for Water Suply and Sanitation.
9. **Water_source_cod:** source of the drinking-water available in the subjects´s household coded. Code 0= Unimproved water source. Code 1= Improved water source.
10. **Sanitation_facility:** type of the sanitation facility available in the study household. Sanitation is considered improved if any of the following facilities is available in the household a) composting toilet; b) ventilated improved pit latrine; c) pit latrine with slab; d) flush/pour to piped sewer system; e) flush/pour to septic tank; or f) flush/pour to pit latrine. Sanitation is considered unimproved if the facility available in the household is a) pit latrine without slab or platform; b) hanging latrine; c) bucket latrine; d) shared or public facilities of any kind; or e) open defecation (disposal of feces in open spaces, open bodies of water or with solid waste). As described in the sanitation and drinking-water ladders of the WHO/UNICEF Joint Monitoring Programme (JMP) for Water Suply and Sanitation.
11. **Sanitation_cod:** characteristics of the sanitation available in the study household. Code 1= improved. Code 2= unimproved.
12. **Date_N_0:** date of baseline anthropometric measurements.
13. **Weight_0:** weight of the study subject measured in kilograms at baseline (before first PC intervention).
14. **Height_0:** height of the study subject measured in centimeters at baseline (before first PC intervention).
15. **WHZ_0:** Weight-for-Height z-score (WHZ) of each participant child < 5 years old, at baseline (before the first PC round), calculated from the anthropometric measurements, using WHO Anthro and WHO software (Department of Nutrition, World Health Organization; Geneva, Switzerland).
16. **low_WHZ_0:** WHZ <-2 SD from the international reference median value, according to the WHO recommendations on z-scores interpretation. At baseline. Code 0= WHZ > -2. Code 1= WHZ < -2 (wasting).
17. **HAZ_0:** Height-for-Age z-score (HAZ) of each participant child, at baseline (before the first PC round), calculated from the anthropometric measurements using WHO Anthro and WHO Anthro Plus softwares (Department of Nutrition, World Health Organization; Geneva, Switzerland).
18. **low_HAZ_0:** HAZ <-2 SD from the international reference median value, according to the WHO recommendations on z-scores interpretation. At baseline. Code 0= HAZ > -2. Code 1= HAZ < -2 (stunting).
19. **WAZ_0:** Weight-for-Age z-score (WAZ) of each participant child, at baseline (before the first PC round), calculated from the anthropometric measurements using WHO Anthro and WHO software (Department of Nutrition, World Health Organization; Geneva, Switzerland).
20. **low_WAZ_0:** WAZ <-2 SD from the international reference median value, according to the WHO recommendations on z-scores interpretation. At baseline. Code 0= WAZ > -2. Code 1= WAZ < -2 (underweight).
21. **Date_N_1:** date of first follow up anthropometric measurements.
22. **Weight_1:** weight of the study subject measured in kilograms at first follow up (before second PC intervention).
23. **Height_1:** height of the study subject measured in centimeters at fist follow up (before second PC intervention).
24. **WHZ_1:** Weight-for-Height z-score (WHZ) of each participant child < 5 years old, at first follow up (before the second PC round), calculated from the anthropometric measurements, using WHO Anthro and WHO software (Department of Nutrition, World Health Organization; Geneva, Switzerland).
25. **low_WHZ_1:** WHZ <-2 SD from the international reference median value, according to the WHO recommendations on z-scores interpretation. At first follow up. Code 0= WHZ > -2. Code 1= WHZ < -2 (wasting).
26. **HAZ_1:** Height-for-Age z-score (HAZ) of each participant child, at first follow up (before the second PC round), calculated from the anthropometric measurements using WHO Anthro and WHO Anthro Plus softwares (Department of Nutrition, World Health Organization; Geneva, Switzerland).
27. **low_HAZ_1**: HAZ <-2 SD from the international reference median value, according to the WHO recommendations on z-scores interpretation. At first follow up. Code 0= HAZ > -2. Code 1= HAZ < -2 (stunting).
28. **WAZ_1 :** Weight-for-Age z-score (WAZ) of each participant child, at first follow up (before the second PC round), calculated from the anthropometric measurements using WHO Anthro and WHO software (Department of Nutrition, World Health Organization; Geneva, Switzerland).
29. **low_WAZ_1:** WAZ <-2 SD from the international reference median value, according to the WHO recommendations on z-scores interpretation. At first follow up. Code 0= WAZ > -2. Code 1= WAZ < -2 (underweight).
30. **Date_N_2:** date of second follow up anthropometric measurements.
31. **Weight_2:** weight of the study subject measured in kilograms at second follow up (before third PC intervention).
32. **Height_2:** height of the study subject measured in centimeters at second follow up (before third PC intervention).
33. **WHZ_2:** Weight-for-Height z-score (WHZ) of each participant child < 5 years old, at second follow up (before the third PC round), calculated from the anthropometric measurements, using WHO Anthro and WHO software (Department of Nutrition, World Health Organization; Geneva, Switzerland).
34. **low_WHZ_2:** WHZ <-2 SD from the international reference median value, according to the WHO recommendations on z-scores interpretation. At second follow up. Code 0= WHZ > -2. Code 1= WHZ < -2 (wasting).
35. **HAZ_2:** Height-for-Age z-score (HAZ) of each participant child, at second follow up (before the third PC round), calculated from the anthropometric measurements using WHO Anthro and WHO Anthro Plus softwares (Department of Nutrition, World Health Organization; Geneva, Switzerland).
36. **low_HAZ_2**: HAZ <-2 SD from the international reference median value, according to the WHO recommendations on z-scores interpretation. At second follow up. Code 0= HAZ > -2. Code 1= HAZ < -2 (stunting).
37. **WAZ_2 :** Weight-for-Age z-score (WAZ) of each participant child, at second follow up (before the third PC round), calculated from the anthropometric measurements, using WHO Anthro and WHO software (Department of Nutrition, World Health Organization; Geneva, Switzerland).
38. **low_WAZ_2:** WAZ <-2 SD from the international reference median value, according to the WHO recommendations on z-scores interpretation. At secondfollow up. Code 0= WAZ > -2. Code 1= WAZ < -2 (underweight).
39. **Date_B_0:** date of baseline blood sample extraction and complete blood count test.
40. **WC_0:** baseline white cells count.
41. **Hgb_0:** baseline hemoglobin value.
42. **Anemia_0:** baseline diagnosis of anemia according to WHO/UNICEF thresholds of hemoglobin value for age and gender. Code 0= not anemic. Code 1= anemic.
43. **E_0:** percentage of eosinophil in the baseline complete blood count
44. **AE_0:** eosinophil absolute count in the baseline complete blood count.
45. **Eosinophilia_0:** baseline diagnosis of eosinophilia defined as eosinophil absolute count above 500 mm^3^. Code 0= eosinophil count < 500. Code 1= eosinophil count > 500.
46. **NIE_0:** Result of NIE-ELISA baseline test for the diagnosis of *Strongyloides stercoralis.* Code 0= negative. Code 1= positive.
47. **Date_B_1:** date of first follow up blood sample extraction and complete blood count test.
48. **WC_1:** first follow up white cells count.
49. **Hgb_1:** first follow up hemoglobin value.
50. **Anemia_1:** first follow up diagnosis of anemia according to WHO/UNICEF thresholds of hemoglobin value for age and gender. Code 0= not anemic. Code 1= anemic.
51. **E_1:** percentage of eosinophil in the first follow up complete blood count
52. **AE_1:** eosinophil absolute count in the first follow up complete blood count.
53. **Eosinophilia_1:** first follow up diagnosis of eosinophilia defined as eosinophil absolute count above 500 mm^3^. Code 0= eosinophil count < 500. Code 1= eosinophil count > 500.
54. **NIE_1:** Result of NIE-ELISA first follow up test for the diagnosis of *Strongyloides stercoralis.* Code 0= negative. Code 1= positive.
55. **Date_B_2:** date of second follow up blood sample extraction and complete blood count test.
56. **WC_2:** second follow up white cells count.
57. **Hgb_2:** second follow up hemoglobin value.
58. **Anemia_2:** second follow up diagnosis of anemia according to WHO/UNICEF thresholds of hemoglobin value for age and gender. Code 0= not anemic. Code 1= anemic.
59. **E_2:** percentage of eosinophil in the second follow up complete blood count
60. **AE_2:** eosinophil absolute count in the second follow up complete blood count.
61. **Eosinophilia_2:** second follow up diagnosis of eosinophilia defined as eosinophil absolute count above 500 mm^3^. Code 0= eosinophil count < 500. Code 1= eosinophil count > 500.
62. **NIE_2:** Result of NIE-ELISA second follow up test for the diagnosis of *Strongyloides stercoralis.* Code 0= negative. Code 1= positive.
63. **Date_P_0: Date of baseline stool sample**
64. **Alum_ CONC_0 :** Ascaris lumbricoides found in the sedimentation/concentration study at baseline. Code 0= negative. Code= 1= positive. Code blank= without data.
65. **Gint_CONC_0 :** Giardia intestinalis found in the sedimentation/concentration study at baseline. Code 0= negative. Code= 1= positive. Code blank= without data.
66. **Encoli_CONC_0:** Entoameba coli found in the sedimentation/concentration study at baseline. Code 0= negative. Code= 1= positive. Code blank= without data.
67. **HKW_CONC_0:** Hookworm found in the sedimentation/concentration study at baseline. Code 0= negative. Code= 1= positive. Code blank= without data.
68. **Stst_CONC_0:** Strongyloides stercoralis found in the sedimentation/concentration study at baseline. Code 0= negative. Code= 1= positive. Code blank= without data.
69. **Hynana_CONC_0:** Hymenolepis nana found in the sedimentation/concentration study at baseline. Code 0= negative. Code= 1= positive. Code blank= without data.
70. **Trtr_CONC_0:** Trichuris trichiura found in the sedimentation/concentration study at baseline. Code 0= negative. Code= 1= positive. Code blank= without data.
71. **Ever_CONC_0:** Enterobius vermicularis found in the sedimentation/concentration study at baseline. Code 0= negative. Code= 1= positive. Code blank= without data.
72. **Taenia_CONC_0:** Taenia found in the sedimentation/concentration study at baseline. Code 0= negative. Code= 1= positive. Code blank= without data.
73. **Stst_HM_0:** Strongyloides stercoralis found in the baseline Harada-Mori study. Code 0= negative. Code= 1= positive. Code blank= without data.
74. **Aduo_HM_0:** Ancylostoma duodenale found in the baseline Harada-Mori study. Code 0= negative. Code= 1= positive. Code blank= without data.
75. **Namer_HM_0:** Necator americanus found in the baseline Harada-Mori study. Code 0= negative. Code= 1= positive. Code blank= without data.
76. **Ever_HM_0:** Enterobius vermicularis found in the baseline Harada-Mori study. Code 0= negative. Code= 1= positive. Code blank= without data.
77. **Stst_BM_0**: Strongyloides stercoralis found in the baseline Baermann study. Code 0= negative. Code= 1= positive. Code blank= without data.
78. **Aduo_BM_0:** Ancylostoma duodenale found in the baseline Baermann study. Code 0= negative. Code= 1= positive. Code blank= without data.
79. **Namer_BM_0:** Necator americanus found in the baseline Baermann study. Code 0= negative. Code= 1= positive. Code blank= without data.
80. **Stst_PA_0**: Strongyloides stercoralis found in the baseline Agar plate study. Code 0= negative. Code= 1= positive. Code blank= without data.
81. **Aduo_PA_0**: Ancylostoma duodenale found in the baseline Agar plate study. Code 0= negative. Code= 1= positive. Code blank= without data.
82. **Namer_PA_0** : Necator americanus found in the baseline Agar plate study. Code 0= negative. Code= 1= positive. Code blank= without data.
83. **Alum_MM_0:** Ascaris lumbricoides found in the baseline McMaster study. Code 0= negative. Code= 1= positive. Code blank= without data.
84. **HKW_MM_0** : Hookworm found in the baseline McMaster study. Code 0= negative. Code= 1= positive. Code blank= without data.
85. **Hynana_MM_0:** Hymenolepis nana found in the baseline McMaster study. Code 0= negative. Code= 1= positive. Code blank= without data.
86. **Trtr_MM_0:** Trichuris trichiura found in the baseline McMaster study. Code 0= negative. Code= 1= positive. Code blank= without data.
87. **Ever_MM_0**: Enterobius vermicularis found in the baseline McMaster study. Code 0= negative. Code= 1= positive. Code blank= without data.
88. **Taenia_MM_0:** Taenia found in the baseline McMaster study. Code 0= negative. Code= 1= positive. Code blank= without data.
89. **Encoli_R_0:** Entoameba coli found by any parasitological method at baseline. Code 0= negative. Code= 1= positive. Code blank= without data.
90. **Gint_R_0** : Giardia intestinalis found by any parasitological method at baseline. Code 0= negative. Code= 1= positive. Code blank= without data.
91. **Alum_R_0:** Ascaris lumbricoides found by any parasitological method at baseline. Code 0= negative. Code= 1= positive. Code blank= without data.
92. **Stst_R_Par_0:** Strongyloides stercoralis found by any parasitological method at baseline. Code 0= negative. Code= 1= positive. Code blank= without data.
93. **Stst_R_Par_NIE_0** : Strongyloides stercoralis found by any parasitological method and/or by NIE ELISA test at baseline. Code 0= negative. Code= 1= positive. Code blank= without data.
94. **Aduo_R_0:** Ancylostoma duodenale found by any parasitological method at baseline. Code 0= negative. Code= 1= positive. Code blank= without data.
95. **Namer_R_0**: Necator americanus found by any parasitological method at baseline. Code 0= negative. Code= 1= positive. Code blank= without data.
96. **Trtr_R_0:** Trichuris trichuira found by any parasitological method at baseline. Code 0= negative. Code= 1= positive. Code blank= without data.
97. **Ever_R_0:** Enterobius vermicularis found by any parasitological method at baseline. Code 0= negative. Code= 1= positive. Code blank= without data.
98. **Hynana_R_0:** Hymenolepis nana found by any parasitological method at baseline. Code 0= negative. Code= 1= positive. Code blank= without data.
99. **HKW_R_0** : Hookworm found by any parasitological method at baseline. Code 0= negative. Code= 1= positive. Code blank= without data.
100. **STH_R_Par_0:** Any STH infection found by any parasitological method at baseline. Code 0= negative. Code= 1= positive. Code blank= without data.
101. **STH_R_Par_NIE_0** : Any STH infection found by any parasitological method and/or by NIE-ELISA test at baseline. Code 0= negative. Code= 1= positive. Code blank= without data.

**Variables ended _1 correspond to first follow up results and variables ended _2 correspond to second follow up results**
